# Artificial intelligence in colposcopic examination: A promising tool to assist junior colposcopists

**DOI:** 10.3389/fmed.2023.1060451

**Published:** 2023-03-15

**Authors:** Aiyuan Wu, Peng Xue, Guzhalinuer Abulizi, Dilinuer Tuerxun, Remila Rezhake, Youlin Qiao

**Affiliations:** ^1^The Affiliated Cancer Hospital of Xinjiang Medical University, Urumqi, China; ^2^School of Population Medicine and Public Health, Peking Union Medical College, Chinese Academy of Medical Sciences, Beijing, China

**Keywords:** artificial intelligence, cervical cancer, colposcopy, diagnostic accuracy, biopsy

## Abstract

**Introduction:**

Well-trained colposcopists are in huge shortage worldwide, especially in low-resource areas. Here, we aimed to evaluate the Colposcopic Artificial Intelligence Auxiliary Diagnostic System (CAIADS) to detect abnormalities based on digital colposcopy images, especially focusing on its role in assisting junior colposcopist to correctly identify the lesion areas where biopsy should be performed.

**Materials and methods:**

This is a hospital-based retrospective study, which recruited the women who visited colposcopy clinics between September 2021 to January 2022. A total of 366 of 1,146 women with complete medical information recorded by a senior colposcopist and valid histology results were included. Anonymized colposcopy images were reviewed by CAIADS and a junior colposcopist separately, and the junior colposcopist reviewed the colposcopy images with CAIADS results (named CAIADS-Junior). The diagnostic accuracy and biopsy efficiency of CAIADS and CAIADS-Junior were assessed in detecting cervical intraepithelial neoplasia grade 2 or worse (CIN2+), CIN3+, and cancer in comparison with the senior and junior colposcipists. The factors influencing the accuracy of CAIADS were explored.

**Results:**

For CIN2 + and CIN3 + detection, CAIADS showed a sensitivity at ~80%, which was not significantly lower than the sensitivity achieved by the senior colposcopist (for CIN2 +: 80.6 vs. 91.3%, *p* = 0.061 and for CIN3 +: 80.0 vs. 90.0%, *p* = 0.189). The sensitivity of the junior colposcopist was increased significantly with the assistance of CAIADS (for CIN2 +: 95.1 vs. 79.6%, *p* = 0.002 and for CIN3 +: 97.1 vs. 85.7%, *p* = 0.039) and was comparable to those of the senior colposcopists (for CIN2 +: 95.1 vs. 91.3%, *p* = 0.388 and for CIN3 +: 97.1 vs. 90.0%, *p* = 0.125). In detecting cervical cancer, CAIADS achieved the highest sensitivity at 100%. For all endpoints, CAIADS showed the highest specificity (55–64%) and positive predictive values compared to both senior and junior colposcopists. When CIN grades became higher, the average biopsy numbers decreased for the subspecialists and CAIADS required a minimum number of biopsies to detect per case (2.2–2.6 cut-points). Meanwhile, the biopsy sensitivity of the junior colposcopist was the lowest, but the CAIADS-assisted junior colposcopist achieved a higher biopsy sensitivity.

**Conclusion:**

Colposcopic Artificial Intelligence Auxiliary Diagnostic System could assist junior colposcopists to improve diagnostic accuracy and biopsy efficiency, which might be a promising solution to improve the quality of cervical cancer screening in low-resource settings.

## 1. Introduction

Cervical cancer remains the fourth most common malignant cancer among women, with an estimated 600,000 new cases and 340,000 deaths in 2020 (1). China has a large population and contributes to nearly 18% (106,000) of new cervical cancer cases and 14% (48,000) of deaths (2), and the morbidity and mortality of cervical cancer tended to increase from 2000 to 2016 in China (3). In 2018, the World Health Organization (WHO) called for global action to eliminate cervical cancer (4), while there is a considerable gap between the WHO goals and the real situation regarding cervical cancer prevention and control in China. Although different human papillomavirus (HPV) vaccines have been approved since 2016 in China, screening is still an indispensable prevention strategy in this post-vaccination era. HPV test has high sensitivity, reproducibility, long-term (at least 5years) reassurance after a negative HPV result, and has been proved as feasible on self-collected samples (5–7). Thus, HPV testing has been widely used in primary cervical cancer screening in many countries, and recommended as the main screening method in the latest WHO guidelines (8).

The application of such a highly sensitive screening method, if not appropriately triaged by another test, will inevitably lead to a much higher colposcopy referral rate. The colposcopic examination is the crucial step linking the primary screening and the histological diagnosis that determines the clinical decision about the optimal management of abnormal lesions (9). Colposcopy plays an irreplaceable role in the precise localization of the biopsy sites and in the early diagnosis of precancerous lesions to reduce the incidence of cervical cancer (10, 11). The accuracy of colposcopy is highly operator-dependent, resulting in low reproducibility and varied diagnostic performance between different resource settings (12). Many low- and middle-income countries are facing the challenges of a shortage of experienced colposcopists, regular colposcopy training courses, a uniform diagnostic standard and strict quality control process, making colposcopy a bottleneck problem that restricts the benefits of cervical cancer screening program (13).

In recent years, artificial intelligence (AI) has been rapidly developed and applied in different fields (14–16). In healthcare, AI has shown promising application value in enhancing diagnosis and personalizing treatment (17–20). There is an increasing interest in the use of deep learning-based AI technologies for the automatic assessment of medical images, which contributes to improving diagnostic accuracy and objectivity and reduces the workload of healthcare workers (21). Such advances also offer the opportunity to tackle the aforementioned challenges in colposcopic diagnosis in cervical cancer screening (22). Xue et al. developed a Colposcopic Artificial Intelligence Auxiliary Diagnostic System (CAIADS) that was trained, tuned, and validated using a large number of colposcopic images and clinical information from 19,435 patients, revealing its potential in improving the diagnostic quality of colposcopy and biopsy in the detection of cervical precancer/cancer (23). In 2022, Zhao et al. (24) concluded that the CAIADS had a higher sensitivity and similar specificity compared with colposcopists. However, the usefulness of the CAIADS in assisting less-experienced colposcopists in clinical practice is unclear.

In this study, we used hospital-based data to further evaluate the diagnostic performance of the CAIADS and its role in assisting junior colposcopists to identify the lesion areas and guide targeted biopsies.

## 2. Materials and methods

### 2.1. Study population

**Figure 1:**
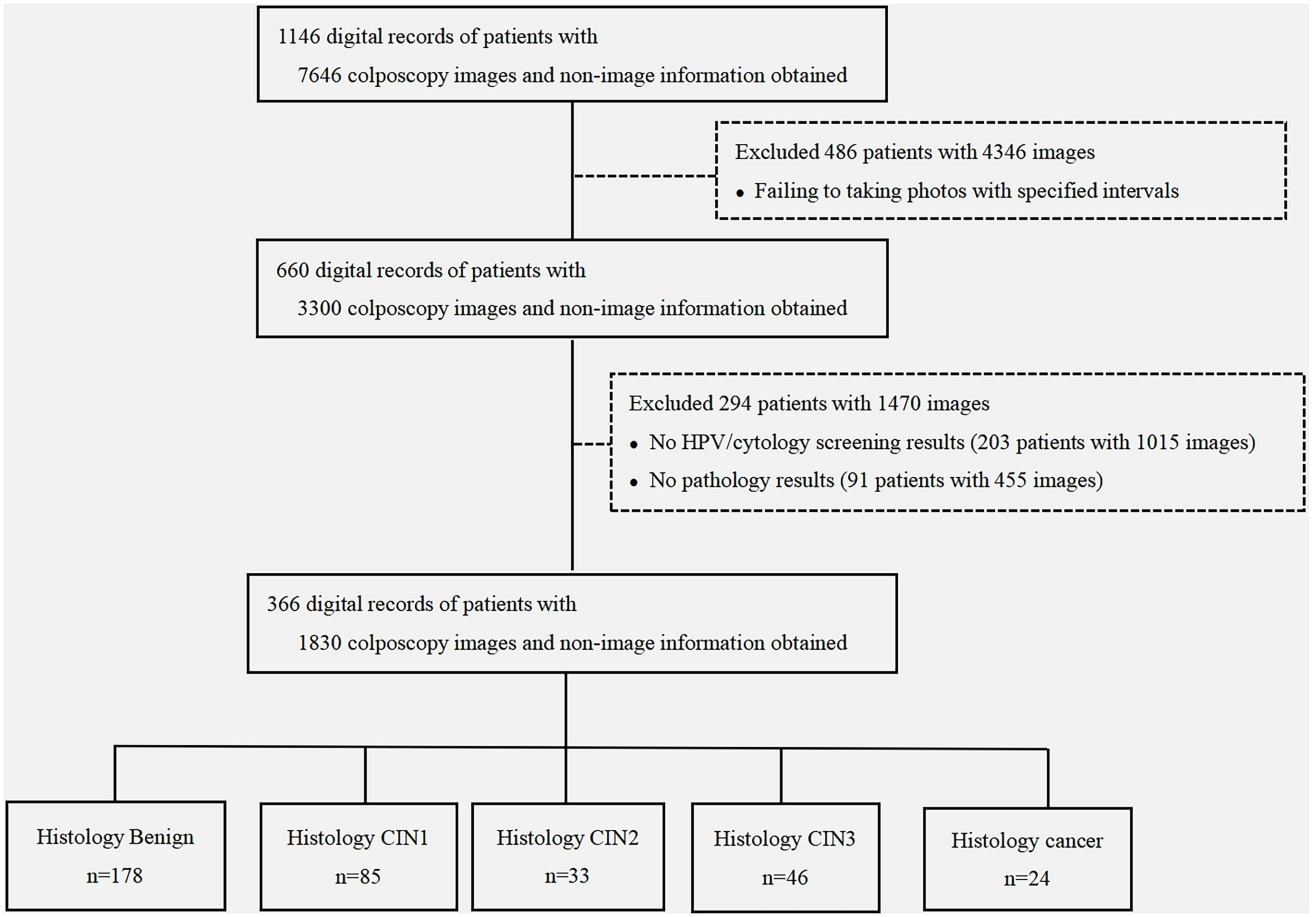
Study flowchart. Non-imaging information comprised the clinical characteristics [human papillomavirus (HPV) status, cytological findings, colposcopic impression, and biopsy results] and demographic characteristics (age, educational level, reproductive history, and menopausal stage) obtained from the medical records. CIN, cervical intraepithelial neoplasia; HPV, human papillomavirus; CIN1, CIN grade 1; CIN2, CIN grade 2; CIN3, CIN grade 3.

This was a hospital-based retrospective study. A total of 1,146 women visited the colposcopy clinics at the Affiliated Cancer Hospital of Xinjiang Medical University in Xinjiang, China, due to abnormal HPV or cytological results or other gynecological symptoms between September 2021 and January 2022. The study cohort comprised women who had standard colposcopic images consecutively taken at 0, 30, 60, 90, and 120 s during the colposcopic examination and had a valid histologic diagnosis. The exclusion criteria were radiotherapy or chemotherapy, lack of definitive pathology results, invalid colposcopic images, unknown HPV status, or unknown cytological information (Figure 1). The digital records of patients, including HPV and cytological information, colposcopic images, type of transformation zone, colposcopic diagnosis by a senior colposcopist, biopsy information (number and site), and histopathological diagnosis were collected from the hospital registry system. General information (age, smoking status, reproductive history, and HPV vaccination status) was collected from the electronic outpatient records. The study was approved by the Ethics Committee of the Affiliated Cancer Hospital of Xinjiang Medical University (approval number: K-2021055). The need for informed consent was waived because the study used anonymized data that were collected retrospectively.

### 2.2. Human papillomavirus testing and cytology

Human papillomavirus testing was performed using the Hybrid Capture 2 assay (Qiagen, Hilden, Germany). HPV was genotyped using GenoArray Diagnostic Kit (HBGA-21PKG, Hybribio, China), which can identify the 21 HPV subtypes, comprising 14 high-risk HPV types (HPV 16, 18, 31, 33, 35, 39, 45, 51, 52, 56, 58, 59, 66, and 68) and seven low-risk HPV types (HPV 6, 11, 42, 43, 44, 53, and 81). HPV results were classified as negative, HPV 16/18-positive, or positive to other high-risk subtypes.

Experienced cytologists from the Affiliated Cancer Hospital of Xinjiang Medical University performed liquid-based cytology (SurePath, BD Oncolarity, United States) and interpreted the results using the Bethesda 2001 classification system (25). Cytological results were classified as negative intraepithelial lesions or malignancies (NILM), atypical squamous cells of undetermined significance (ASC-US), atypical squamous cells which cannot exclude high-grade squamous intraepithelial lesion (ASC-H), low-grade squamous intraepithelial lesions (LSIL), and high-grade squamous intraepithelial lesions (HSIL) or worse (including squamous cell carcinoma, adenocarcinoma *in situ*, and glandular abnormalities).

### 2.3. Colposcopic procedure and histological confirmation

A senior colposcopist with over 20 years of specialized experience in the colposcopy clinic used a high-resolution electronic colposcope (EDAN, China) to perform the colposcopic examination in accordance with standard clinical guidelines (26). In brief, 5% acetic acid was applied to the cervix, and the visibility of the squamocolumnar junction, presence of aceto-whitening, and colposcopic lesions were documented for each woman. The final colposcopic diagnosis was recorded as benign/normal, low-grade lesion, or high-grade lesion. The colposcopic images were saved in JPEG format (640 × 480 pixels). For each woman, the colposcopic images consisted of five sequential images, namely a pre-acid image (at 0 s) and four post-acid images with an approximate time interval of 30 s (i.e., at 60, 90, 120, and 150 s) (23). Direct biopsy was performed when the colposcopic impression was satisfactory and suspicious lesions were seen; if the colposcopy was unsatisfactory or the result was HPV 16/18-positive or cytology showed high-grade abnormalities, four random biopsies were taken at the 3, 6, 9, and 12 o’clock positions.

Senior pathologists in the Affiliated Cancer Hospital of Xinjiang Medical University performed the histologic diagnosis using hematoxylin and eosin-stained slides. When the lesions were equivocal, p16 and Ki67 immunohistochemical staining of the tissue specimens was performed and the final diagnosis was made after a conjunctive analysis of the slides stained with hematoxylin and eosin and p16/Ki67. All histopathological findings were categorized by the cervical intraepithelial neoplasia (CIN) classification system as benign, CIN grades 1, 2, and 3, and cancer, with the worst finding used as the final diagnosis.

In addition to the examinations described earlier, a junior colposcopist with 1 year of experience working in the gynecological department reviewed all colposcopic images. The junior colposcopist was aware of the HPV status and cytological findings but was blinded to the colposcopic diagnosis by the senior colposcopist and the histological diagnosis. The junior colposcopist categorized the colposcopic findings using the 2014 WHO Classification of Female Reproductive System Neoplasms (27) as normal/benign, LSIL, HSIL, and cancer.

### 2.4. Diagnosis by the CAIADS

The CAIADS that was developed and initially validated by Xue et al. (23) was used to diagnose the cervical lesions. In brief, both the colposcopic images and the non-imaging information (cytology and HPV status) were inputted into the CAIADS to enable it to make a diagnostic judgment. The CAIADS algorithm mapped the input features (colposcopic images and non-imaging information) to the corresponding two target tasks (grading of the colposcopic impressions and guidance of biopsies) based on four deep learning networks, namely cervix detection, feature encoding, graph convolutional network-based feature fusion, and lesion area segmentation networks (23, 28).

The findings of the CAIADS were categorized into three groups: benign, LSIL, and HSIL or worse (HSIL+), and the biopsy number and specific sites were indicated by the system with blue circles (Supplementary Figure 1). The CAIADS and the junior colposcopist received the same anonymized colposcopic images and non-imaging data (cytology and HPV status) to make an independent diagnosis while blinded to the senior colposcopist’s findings and the histological results. To evaluate the role of the CAIADS in assisting the junior colposcopist, the order of the colposcopic records was changed and the junior colposcopist performed a second review with the knowledge of the CAIADS results; these findings were defined as the CAIADS-assisted junior colposcopist (abbreviated as CAIADS-Junior in the subsequent text). The junior colposcopist also indicated the biopsy sites and number of biopsies on the original colposcopic images with and without the knowledge of the CAIADS.

### 2.5. Statistical analysis

The demographic and clinical characteristics were summarized using descriptive statistics. Taking the histological diagnosis as the gold standard, the diagnostic performances of the different subspecialists (CAIADS, senior colposcopist, junior colposcopist, and CAIADS-Junior) were evaluated separately for the different histology endpoints (CIN2 +, CIN3 +, and cancer). The Wilson score approach was used to calculate the sensitivity, specificity, positive predictive value, and negative predictive value with 95% confidence intervals (95% CIs). The sensitivity and specificity of the subspecialists were compared using McNemar’s test. The areas under the curves (AUCs) were compared using the DeLong test (29). To evaluate the biopsy efficacy, the number of captured biopsies required per case (BNR) was calculated for each histology endpoint and the biopsy sensitivity was calculated (number of biopsies indicated by the subspecialists/the total number of diagnosed biopsies for specific endpoints). Binary logistic regression was used to estimate the odds ratios and 95% CIs to assess their impact on the CAIADS regarding accurate diagnosis and underdiagnosis. Age, ethnicity, BMI, educational level, parity, stage of menopause, cytological result, HPV status, and biopsy type were analyzed as the demographic and clinical characteristics potentially influencing the diagnostic accuracy and underdiagnosis of CAIADS. The accurate diagnosis was defined as the conditions in which the CAIADS indicated an abnormality (LSIL +) and histology confirmed CIN2 + or when the CAIADS judged a lesion as normal without the need for biopsy and histology confirmed the lesion as < CIN2; all other conditions were regarded as an inaccurate diagnosis. Among women diagnosed as normal by the CAIADS, histological confirmation of CIN2 + was defined as underdiagnosis, while a histological confirmation of normal was defined as no underdiagnosis.

Statistical significance was defined as *p* < 0.05 (two-sided). All analyses were performed using IBM SPSS version 28 (IBM, New York, NY, United States) and MedCalc Statistical Version 20 (MedCalc Software Ltd., Ostend, Belgium).

## 3. Results

Figure 1 shows the flowchart of the selection of the study population. The medical records of 1,146 women with 7,646 colposcopic images were reviewed. Among them, 660 women with complete colposcopic images (five images per woman with an approximately 30-s interval between images) were identified, resulting in a total of 3,300 colposcopic images. Two-hundred-and-ninety-four women were excluded due to incomplete clinical information. A total of 366 women with a median age of 44 years (range 22–85 years, interquartile range 36–52 years) with 1,830 colposcopic images were included in the final analysis (Figure 1). The detailed demographic information of the cohort is presented in Table 1.

**Table 1:** Demographic characteristics of the study population.

Characteristics	*N*	%
Age (years)		
Mean ± SD	44 ± 10.5	/
Middle (IQR)	44(36–52)	/
BMI (kg/m^2^)		
Mean ± SD	23.9 ± 3.4	/
Middle (IQR)	23.5(21.6–25.7)	/
Ethnicity		
Han	215	58.7
Uyghur	110	30.1
Other^*^	41	11.2
Education		
Primary school or less	56	15.3
Middle school	81	22.1
High school	69	18.9
Graduate	160	43.7
Age of the first parturition (years)		
Mean ± SD	25 ± 4.1	/
Middle (IQR)	24(22–27)	/
Contraception		
No contraception	172	47
Condom	95	26
Oral contraceptive	4	1.1
Intrauterine devices	89	24.3
Sterilization	6	1.6
Number of pregnancies		
≤2	147	40.2
(3–5)	192	52.5
≥6	27	7.4
Number of parities		
≤1	189	51.6
>1	177	48.4
Stages of menopause		
Postmenopausal	113	30.9
Pre-menopausal	253	69.1

* Kazak, Mongolian, Hui, and Kirgiz.

SD, standard deviation; IQR, interquartile range.

### 3.1. Clinical characteristics and colposcopic findings determined by the senior colposcopist

As shown in Table 2, 145 (39.6%) women had cytological results that showed atypical squamous cells of undetermined significance or worse, and 308 women (84.2%) tested positive for HPV, of which 164 (44.8%) were HPV 16/18-positive. The histology results were: 178 normal lesions (48.6%), 85 CIN1 cases (23.2%), 33 CIN2 cases (9.0%), 46 CIN3 cases (12.6%), and 24 cancer cases (6.6%). Most women (87.7%) were assessed as having an adequate cervical impression by the senior colposcopist. The senior colposcopist classified the colposcopic findings as benign (*n* = 132, 36.1%), low-grade lesions (*n* = 139, 38.0%), and high-grade lesions or worse (*n* = 95, 26.0%).

**Table 2:** Human papillomavirus (HPV), cytological, and colposcopic findings by histological diagnosis.

	Total (*N*)	Benign, *n* (%)		CIN1, *n* (%)		CIN2, *n* (%)		CIN3, *n* (%)		Cancer, *n* (%)	
	366	178	48.6	85	23.2	33	9	46	12.6	24	6.6
Cytology											
NILM	221	127	57.5	59	26.7	10	4.5	16	7.2	9	4.1
ASC-US	63	28	44.4	13	20.6	8	12.7	10	15.9	4	6.3
LSIL	36	15	41.7	11	30.6	5	13.9	4	11.1	1	2.8
ASC-H	14	3	21.4	0	0	4	28.6	4	28.6	3	21.4
HSIL	30	4	13.3	1	3.3	6	20	12	40	7	23.3
AGC	2	1	50	1	50	0	0	0	0	0	0
HPV test											
Negative	58	39	67.2	15	25.9	0	0	3	5.2	1	1.7
HPV 16/18+	164	64	39	25	15.2	20	12.2	34	20.7	21	12.8
Other hrHPV+	144	75	52.1	45	31.3	13	9	9	6.3	2	1.4
General assessment by senior colposcopist											
Adequate	321	152	47.4	73	22.7	33	10.3	45	14	18	5.6
Inadequate	45	26	57.8	12	26.7	0	0	1	2.2	6	13.3
Transformation zone by Senior											
Fully visible	245	115	46.9	55	22.4	25	10.2	37	15.1	13	5.3
Partially visible	83	41	49.4	19	22.9	8	9.6	8	9.6	7	8.4
Not visible	38	22	57.9	11	28.9	0	0	1	2.6	4	10.5
Colposcopy findings by the senior colposcopist											
Benign	132	98	74.2	25	18.9	2	1.5	5	3.8	2	1.5
LSIL	139	67	48.2	48	34.5	11	7.9	10	7.2	3	2.2
HSIL	75	11	14.7	12	16	19	25.3	28	37.3	5	6.7
Cancer	20	2	10	0	0	1	5	3	15	14	70
Biopsy types performed by the senior colposcopist											
Targeted biopsy	248	92	37.1	63	25.4	31	12.5	40	16.1	22	8.9
Random biopsy	118	86	72.9	22	18.6	2	1.7	6	5.1	2	1.7
No. of biopsy performed by the senior colposcopist											
Mean ± SD	4 ± 0.9	4	±0.8	4	±0.8	4	±0.8	4	±0.7	3	±1.4
Middle (IQR)	4(3–4)	4	(3–4)	4	(3–4)	4	(4–5)	4	(4–5)	4	(3–4)
Colposcopy findings by CAIADS											
Benign	187	116	62	51	27.3	6	3.2	14	7.5	0	0
LSIL	131	53	40.5	25	19.1	21	16	22	16.8	10	7.6
HSIL+	48	9	18.8	9	18.8	6	12.5	10	20.8	14	29.2
Biopsy indicated by CAIADS											
Not indicated	187	116	62	51	27.3	6	3.2	14	7.5	0	0
Biopsy indicated	179	62	34.6	34	19	27	15.1	32	17.9	24	13.4
No. of biopsy indicated by the CAIADS											
Mean ± SD	1 ± 1.5	1	±1.4	1	±1.5	2	±1.5	2	±1.5	2	±1.3
Middle (IQR)	0(0–3)	0	(0–2)	0	(0–2)	3	(1–3)	2	(0–3)	2	(1–3)
Colposcopy findings by the junior colposcopist											
Benign	149	72	48.3	56	37.6	11	7.4	7	4.7	3	2
LSIL	140	81	57.9	18	12.9	13	9.3	22	15.7	6	4.3
HSIL	59	23	39	10	16.9	9	15.3	14	23.7	3	5.1
Cancer	18	2	11.1	1	5.6	0	0	3	16.7	12	66.7
Biopsy indicated by the junior colposcopist											
Not indicated	115	72	62.6	22	19.1	11	9.6	7	6.1	3	2.6
Biopsy indicated	251	106	42.2	63	25.1	22	8.8	39	15.5	21	8.4
No. of biopsy indicated by the junior colposcopist											
Mean ± SD	3 ± 1.9	3	±2.1	3	±1.9	4	±1.1	4	±1.3	3	±1.5
Middle (IQR)	4(1–4)	4	(0–5)	4	(1–5)	4	(3–5)	4	(4–4)	3	(1–4)
Colposcopy findings by the CAIADS-Junior											
Benign	132	95	72	32	24.2	3	2.3	1	0.8	1	0.8
LSIL	147	66	44.9	38	25.9	20	13.6	18	12.2	5	3.4
HSIL	71	15	21.1	14	19.7	9	12.7	26	36.6	7	9.9
Cancer	16	2	12.5	1	6.3	1	6.3	1	6.3	11	68.8
Biopsy indicated by the CAIADS-Junior											
Unperformed	95	71	74.7	21	22.1	2	2.1	0	0	1	1.1
Performed	271	107	39.5	64	23.6	31	11.4	46	17	23	8.5
No. of biopsy indicated by the CAIADS-Junior											
Mean ± SD	3 ± 2.0	3	±2.2	3	±2.0	4	±1.1	4	±0.7	2	±1.7
Middle (IQR)	4(0–4)	3	(0–4)	4	(1–4)	4	(3–4)	4	(3–4)	1	(1–4)

CAIADS, Colposcopic Artificial Intelligence Auxiliary Diagnostic System; IQR, interquartile range; NILM, negative for intraepithelial lesion or malignancy; ASC-US, atypical squamous cells of undetermined significance; LSIL, low-grade squamous intraepithelial lesions; HSIL, high-grade squamous intraepithelial lesions; ASC-H, atypical squamous cells of undetermined significance that cannot exclude HSIL; HSIL+, HSIL or worse; hrHPV+, positive for high-risk subtypes; AGC, atypical glandular cells; HPV, human papillomavirus; SD, standard deviation; CIN, cervical intraepithelial neoplasia; CIN1, CIN grade 1; CIN2, CIN grade 2; CIN3, CIN grade 3; CAIADS-Junior: junior colposcopist with the assistance of the CAIADS.

### 3.2. Colposcopic findings of the CAIADS and the junior colposcopist

The CAIADS indicated 131 LSIL cases (35.8%) and 48 HISL + cases (13.1%), whereas the junior colposcopist indicated 140 LSIL cases (38.3%) and 77 HISL + cases (21.0%). When assisted by the CAIADS, the detection rate of the junior colposcopist increased to 40.2% (*n* = 147) for LSIL and 23.8% (*n* = 87) for HSIL +.

### 3.3. Diagnostic performance of the CAIADS compared with different colposcopists

Table 3 and Supplementary Figure 2 show the diagnostic performance of the CAIADS in comparison with the junior and senior colposcopists, and its value in assisting the junior colposcopist. Concerning CIN2 + and CIN3 + detections, the CAIADS showed a sensitivity of approximately 80%, which was not significantly lower than the sensitivity of the senior colposcopist (for CIN2 +: 80.6, 95% CI: 71.9–87.1% vs. 91.3, 95% CI: 84.2–95.3%, *p* = 0.061; for CIN3 +: 80.0, 95% CI: 69.2–87.7 vs. 90.0, 95% CI: 80.7–95.1%, *p* = 0.189). The sensitivity of the junior colposcopist was significantly increased with the assistance of the CAIADS (for CIN2 +: 95.1, 95% CI: 89.1–97.9% vs. 79.6, 95% CI: 70.8–86.3%, *p* = 0.002; for CIN3 +: 97.1, 95% CI: 90.2–99.2% vs. 85.7, 95% CI: 75.7–92.1%, *p* = 0.039). The sensitivity of the CAIADS-Junior was slightly higher than that of the senior colposcopist (for CIN2 +: 95.1, 95% CI: 89.1–97.9% vs. 91.3, 95% CI: 84.2–95.3%, *p* = 0.388; for CIN3+: 97.1, 95% CI: 90.2–99.2% vs. 90.0, 95% CI: 80.7–95.1%, *p* = 0.125) and significantly higher than that of the CAIADS (for CIN2+: 95.1, 95% CI: 89.1–97.9% vs. 80.6, 95% CI: 71.9–87.1%, *p* = 0.003; for CIN3+: 97.1, 95% CI: 90.2–99.2% vs. 80.0, 95% CI: 69.2–87.7%, *p* = 0.004). In detecting cervical cancer, the CAIADS achieved the highest sensitivity at 100% and assisted the junior colposcopist to improve the sensitivity from 87.5 to 95.8%, although this difference was not statistically significant (*p* = 0.625).

**Table 3:** Diagnostic performance of the subspecialists for different clinical endpoints.

Subspecialists	Sensitivity	Specificity	PPV	NPV
	(%; *n*/*N*) 95% CI	(%; *n*/*N*) 95% CI	(%; *n*/*N*) 95% CI	(%; *n*/*N*) 95% CI
**CIN2+**				
Senior colposcopist	91.3 (94/103)	46.8 (123/263)	40.2 (94/234)	93.2 (123/132)
	84.2–95.3	40.8–52.8	34.1–46.6	87.6–96.4
CAIADS	80.6 (83/103)	63.5 (167/263)	46.4 (83/179)	89.3 (167/187)
	71.9–87.1	63.0–71.4	39.2–53.7	84.1–93.0
Junior colposcopist	79.6 (82/103)	48.7 (128/263)	37.8 (82/217)	85.9 (128/149)
	70.8–86.3	42.7–54.7	31.6–44.4	79.4–90.6
CAIADS-Junior	95.1 (98/103)	48.3 (127/263)	41.9 (98/234)	96.2 (127/132)
	89.1–97.9	42.3–54.3	35.7–48.3	91.4–98.37
**CIN3^+^**				
Senior colposcopist	90.0 (63/70)	42.2 (125/296)	26.9 (63/234)	94.7 (125/132)
	80.7–95.1	36.7–47.9	21.7–33.0	89.5–97.4
CAIADS	80.0 (56/70)	58.4 (173/296)	31.3 (56/179)	92.5 (173/187)
	69.2–87.7	52.8–63.9	25.0–38.4	87.8–95.5
Junior colposcopist	85.7 (60/70)	47.0 (139/296)	27.6 (60/217)	93.3 (139/149)
	75.7–92.1	41.4–52.7	22.1–34.0	88.1–96.3
CAIADS-Junior	97.1 (68/70)	43.9 (130/296)	29.1 (68/234)	98.5 (130/132)
	90.2–99.2	38.4–49.6	23.2–35.2	94.6–99.6
Cancer				
Senior colposcopist	91.7 (22/24)	38.0 (130/342)	9.4 (22/234)	98.5 (130/132)
	74.2–97.7	33.0–43.3	6.3–13.8	94.6–99.6
CAIADS	100 (24/24)	54.7 (187/342)	13.4 (24/179)	100.0 (187/187)
	86.2–100.0	49.4–59.9	9.2–19.2	97.99–100
Junior colposcopist	87.5 (21/24)	42.7 (146/342)	9.7 (21/217)	98.0 (146/149)
	69.0–95.7	37.6–48.0	6.4–14.3	94.25–99.31
CAIADS-Junior	95.8 (23/24)	38.3 (131/342)	9.8 (23/234)	99.2 (131/132)
	79.8–99.3	33.3–43.6	6.6–14.3	95.8–99.9

CAIADS, Colposcopic Artificial Intelligence Auxiliary Diagnostic System; CAIADS-Junior, CAIADS-assisted junior colposcopist; NPV, negative predictive value; PPV, positive predictive value; CIN, cervical intraepithelial neoplasia; 95% CI, 95% confidence interval; CIN2+, CIN grade 2 or worse; CIN3+, CIN grade 3 or worse; CI: confidence interval.

**Figure 2:**
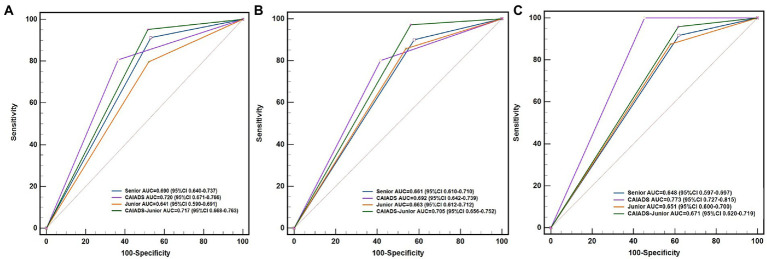
ROCs and AUCs of the subspecialists for the different disease endpoints. **(A)** CIN2+, **(B)** CIN3+, and **(C)** cancer. CAIADS, colposcopic artificial intelligence auxiliary diagnostic system; CAIADS-Junior, CAIADS-assisted junior colposcopist; ROC curve, receiver operator characteristic curve; AUC, area under the curve; CIN, cervical intraepithelial neoplasia; CIN2+, CIN grade 2 or worse; CIN3+, CIN grade 3 or worse.

For all endpoints, the CAIADS showed the highest specificity (55–64%) and the highest positive predictive values compared with the senior and junior colposcopists. Furthermore, the CAIADS had the highest overall accuracy for all endpoints. As shown in Figure 2 and Supplementary Figure 2E, there were significant differences between the AUC of the CAIADS and the junior colposcopist in detecting CIN2 + and cancer, although this difference was not significant for detecting CIN3 +. The AUC of the CAIADS was significantly higher than that of the senior colposcopist in detecting cancer (0.773 vs. 0.648; *p* < 0.001).

### 3.4. Biopsy efficacy and sensitivity of the CAIADS and CAIADS-junior

**Figure 3:**
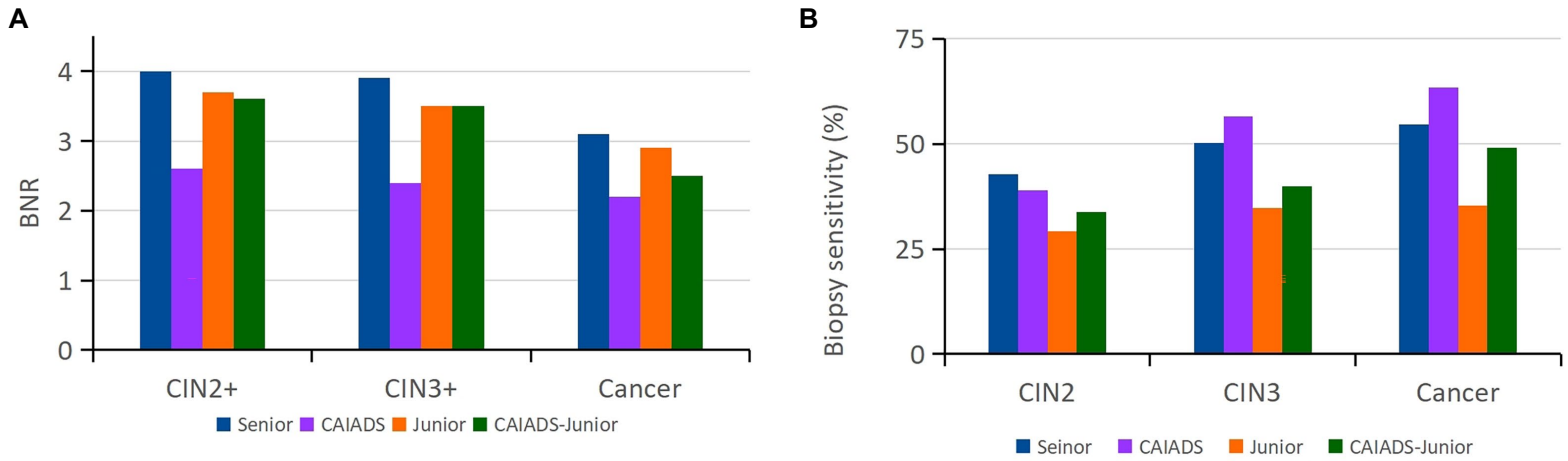
Biopsy efficacy and sensitivity of the subspecialists. **(A)** Number of biopsy sites required to detect each high-grade cervical lesion by the subspecialists. **(B)** Biopsy sensitivity of the subspecialists for each cervical lesion. CAIADS, colposcopic artificial intelligence auxiliary diagnostic system; CAIADS-Junior, CAIADS-assisted junior colposcopist; BNR, number of biopsies needed to detect each case for different endpoints (CIN2+/CIN3+/cancer); CIN, cervical intraepithelial neoplasia; CIN2+, CIN2 or worse; CIN3+, CIN3 or worse.

A total of 1,415 biopsies were taken from the 366 women by the senior colposcopist. To reflect the biopsy efficacy of the subspecialists, the BNRs (Figure 3A) and biopsy sensitivity (Figure 3B) were calculated for different histological lesions. As the lesion grade became more severe, the average number of biopsies required per case decreased for the subspecialists, among which the CAIADS required the lowest number of biopsies per case (2.6 for CIN2 +, 2.4 for CIN3 +, and 2.2 for cancer). The junior colposcopist demonstrated very similar BNRs (3–4) with or without the assistance of the CAIADS. The CAIADS had the highest biopsy sensitivity for CIN3 (56.5, 95% CI: 45.8–66.7%) and for cancer (63.5, 95% CI: 49.8–75.7%). The junior colposcopist showed the lowest biopsy sensitivity for all endpoints (for CIN2: 29.1, 95% CI: 21.7–37.5%; for CIN3: 34.7, 95% CI: 27.8–42.1%; for cancer: 35.4, 95% CI: 24.5–47.5%); these sensitivities were increased with the assistance of the CAIADS (for CIN2: 33.8, 95% CI: 26.1–42.3%; for CIN3: 40.0, 95% CI: 33.1–47.2%; for cancer: 49.1, 95% CI: 36.4–62.0%).

### 3.5. Factors influencing the accuracy of the CAIADS judgement

Table 4 and Supplementary Table 1 show the results of the uni- and multi-variate logistic regression analyses to assess the factors influencing the diagnostic accuracy and underdiagnosis of CAIADS. Multivariate logistic regression showed that parity (> 1) was the only demographic factor that decreased the chance of underdiagnosis by the CAIADS (OR: 0.21, 95% CI: 0.52–0.84; Table 4). No clinical factor was associated with the accuracy of the CAIADS (Supplementary Table 1).

**Table 4:** Logistic regression analysis of the demographic and clinical characteristics affecting CAIADS-related underdiagnosis in detecting cervical diseases.

Characteristic	Univariate Analysis		*p*	Multivariable Analysis	*p*
	*n* (%)	OR (95% CI)		OR (95% CI)	
*Age*			0.056		0.419
[20-49]	104 (55.6)	1	–	1	–
≥ 50	83 (44.4)	2.57 (0.98–6.78)	–	1.85 (0.42–8.22)	–
*Ethnicity*			0.246		0.923
Han	108 (57.8)	1	–	1	**–**
Others^a^	79 (42.2)	0.55 (0.20–1.51)	–	0.94 (0.25–3.52)	–
*BMI*			0.062		0.055
[18.5–23.9]	94 (50.3)	1	–	1	**–**
< 18.5 or > 23.9	93 (49.7)	2.60 (0.95–7.09)	–	3.09 (0.98-9.63)	**–**
*Education*			**0.042**		0.053
Blew middle school	72 (38.5)	**1**	**–**	1	–
High school or above	115 (61.5)	**0.37** **(0.15-0.97)**	**–**	0.26 (0.08-1.09)	–
*Number of parities*			0.069		**0.027**
≤ 1	94 (50.3)	1	–	**1**	**–**
> 1	93 (49.7)	0.39 (0.14-1.08)	–	**0.21** **(0.52-0.84)**	**–**
*Stages of menopause*			0.085		0.730
Postmenopausal	61 (32.6)	1	–	1	–
Pre-menopausal	126 (67.4)	0.44 (0.17–1.12)	–	0.77 (0.18-3.34)	**–**
*Cytology*			0.685		0.734
NILM	105 (56.1)	1	–	1	**–**
ASC-US and LSIL	55 (29.4)	0.71 (0.24–2.10)	0.533	0.85 (0.25–2.92)	0.790
ASC-H, HSIL, and AGC	27 (14.4)	0.57 (0.12-2.68)	0.473	0.52 (0.10-2.71)	0.436
*HPV test*			0.996		0.897
Negative	28 (15.0)	1	–	1	–
HPV 16/18 positive	92 (49.2)	1.02 (0.26–3.98)	0.982	1.08 (0.24–4.86)	0.924
Other high-risk subtypes positive	67 (35.8)	0.97 (0.23–4.07)	0.969	0.82 (0.16–4.07)	0.806
*Biopsy types*	-		**0.018**		0.055
Targeted biopsy	109 (58.3)	**1**	**–**	1	**–**
Random biopsy	78 (41.7)	**0.22** **(0.06–0.77)**	**–**	0.27 (0.07–1.03)	**–**

a Uighur, Kazak, Mongolian, Hui, Kirgiz; *p* < 0.05; significant *p*-values are in bold font.

OR, odds ratio; CI, confidence interval; BMI, body mass index in kg/m^2^; NILM, negative for intraepithelial lesion or malignancy; ASC-US, atypical squamous cells of undetermined significance; LSIL, low-grade squamous intraepithelial lesions; HSIL, high-grade squamous intraepithelial lesions; ASC-H, atypical squamous cells of undetermined significance that cannot exclude HISL; AGC, atypical glandular cells, HPV, human papillomavirus.

## 4. Discussion

Colposcopy is the cornerstone of the cervical cancer screening program and is used in combination with pathology to determine the best management strategy. However, the accuracy of colposcopy is a worldwide concern due to its subjective nature as it is highly operator-dependent; this issue is compounded in low- to middle-income countries with a limited number of well-trained colposcopists. The inaccuracy of colposcopy is reflected by the large variation in the consistency rate between colposcopic findings and pathology, ranging from 37 to 66% (30–34). With the worldwide trend of using HPV testing as the primary screening method, which inevitably leads to a significant increase in colposcopy referrals, there is an increasing demand for high-quality colposcopic examination to precisely identify the cervical lesions and locate the biopsy sites to obtain the final pathological diagnosis. If the accuracy of colposcopy-directed biopsy cannot be guaranteed, the efficacy of the screening program will be limited.

The great advances in AI technology have brought the opportunity to improve medical practice in recent years. AI-based or deep learning-based colposcopic methods have shown promise in several studies (35–38). In these studies, AI-based colposcopy or deep learning-based colposcopy systems were trained and validated using more than 10,000 colposcopic images, and the performances of these systems were compared with colposcopists with different levels of experience. In diagnosing histologically confirmed HSIL + cases, the reported sensitivities of AI-colposcopy, colposcopists, and AI-assisted colposcopists are 74.1–82.8%, 19.5–100%, and 66.7–84.5%, respectively. Overall, the diagnostic performance of colposcopists varies greatly, whereas the sensitivity of AI colposcopy tends to be stable between studies. In our study, the CAIADS and CAIADS-Junior findings had a sensitivity of more than 80% for high-grade lesions. These findings further reflect the fact that as an objective tool that is trained, set up, and validated using thousands of images, AI colposcopy has great potential to ensure the quality of colposcopic examination, which is of particular importance in areas that lack well-trained colposcopists.

The major aim of the colposcopic examination is to precisely obtain biopsies to confirm a histological diagnosis of HSIL or cervical cancer. Most studies have only explored the diagnostic performance of AI-colposcopy (35, 36, 39, 40), while there is a lack of evidence regarding the role of AI in the last critical step (guiding biopsy), which makes AI colposcopy less practical in the areas lacking well-trained colposcopists. The CAIADS used in our study showed its advantages in colposcopic diagnosis, demonstrated its superiority in a colposcopy-targeted directed biopsy, and revealed its potential in assisting junior colposcopists to improve their targeted biopsy performance, achieving a higher efficacy and biopsy sensitivity than that of the junior colposcopist alone.

The accuracy of colposcopic diagnosis and targeted biopsy might be influenced by various factors, such as age, menopause status, cytological abnormalities, HPV infection status, and type of transformation zone (41). We performed univariate and multivariate logistic regression analyses to identify the factors associated with the accuracy of the CAIADS and CAIADS-related underdiagnosis. Our study revealed that the number of parities was negatively correlated with underdiagnosis of the CAIADS, which is consistent with previous evidence that deliveries significantly maintain the transformation zone on the exocervix (42), making it easier for the CAIADS to identify the lesion areas. Overall, the role of the CAIADS is to assist colposcopists rather than supersede colposcopists in clinical practice and decision-making.

External validation of the CAIADS has provided powerful evidence for its accuracy in the colposcopic examination (43). The present study used an independent real-world dataset (neither training nor an adjustment dataset) to evaluate the feasibility and effectiveness of the CAIADS, providing evidence for its clinical application in colposcopy clinics. The CAIADS was first applied in Xinjiang and was applied to ethnically diverse populations, affirming its geographical and ethnic generalization abilities. External validation of the CAIADS identified man–machine cooperation rather than man–machine confrontation. Previous studies have shown that humans and AI achieve similar outcomes and have suggested that humans will be replaced by AI (23, 44, 45). However, the present study revealed that the AI-assisted colposcopist achieved the best results, which is more in line with ethical, moral, and legal requirements than the use of AI alone.

The implementation of the CAIADS still has the following problems in less-developed areas (13, 46). First, the quality of available cervical information (screening data, colposcopy images, etc.) may affect the colposcopic interpretation, and descriptive terms are not standardized in colposcopy practice due to the use of different types of colposcopic equipment, including cervical labeling, annotation, classification, and quality supervision (26, 31, 47). Thus, we aim to apply the CAIADS in various scenarios. Second, a wide area network may be difficult to achieve in less-developed areas due to the requirement for high-definition images and large running memory. Therefore, we aim to develop a software version of the CAIADS that is feasible using a local service network. Finally, colposcopists in low-resource areas may have incorrect notions about AI. For colposcopists to effectively use the CAIAD, it is important to understand that AI is a tool that assists the physician and does not take the place of a physician in making decisions.

The main strengths of this study are that we externally validated AI-based colposcopy (using the CAIADS) in diagnosing cervical lesions and targeting biopsy sites based on a hospital-based retrospective study in Xinjiang, China, proving important evidence on the performance and feasibility of CAIADS in resource-limited areas. While, the major limitation is that, as a retrospective study, the CAIADS and the junior colposcopist made decisions by reviewing high-resolution colposcopic images. Therefore, some potentially malignant cases that were detected by either the CAIADS or the junior colposcopist might have been missed and thus not biopsied by the senior colposcopist. However, the senior colposcopist who performed the colposcopic examinations had more than 20 years of working experience in a colposcopy clinic, which may have reduced the risk of missed cases. Furthermore, only one junior colposcopist with 1 year of experience reviewed the colposcopic images, inevitably leading to observer bias. Given that the current study is one of the very few studies evaluating the role of AI-colposcopy in assisting a junior colposcopist in diagnosing and guiding the biopsy during cervical cancer screening, which might be the most practical way to use AI in the screening setting, the promising findings provide the necessary evidence for future population-based, multicenter studies to further evaluate the use of AI in real-world settings.

## 5. Conclusion

The CAIADS may enhance the diagnostic and biopsy accuracy of junior colposcopists. Therefore, the CAIADS might be a promising solution to improve the colposcopy practice in low-resource areas with limited numbers of well-trained colposcopists.

## Data availability statement

The datasets used and/or analyzed during the current study are available from the corresponding author on reasonable request.

## Ethics statement

The studies involving human participants were reviewed and approved by the Research Ethics Committee of Affiliated Tumor Hospital of Xinjiang Medical University. Written informed consent for participation was not required for this study in accordance with the national legislation and the institutional requirements.

## Author contributions

RR and YQ designed the study. AW, PX, GA, and DT were involved in the administration of fieldwork, data collection, and assembly. AW, PX, and RR participated in manuscript writing, data analysis, and interpretation. YQ and GA provided constructive comments and revisions to the manuscript. All authors contributed to the article and approved the submitted version.

## Funding

This study was supported by the State Key Laboratory of Pathogenesis, Prevention, and Treatment of High Incidence Diseases in Central Asia Fund (SKL-HIDCA-2020-GJ2), the Xinjiang Uygur Autonomous Region Postgraduate Research Innovation Project (XJ2022G162), the Postdoctoral Fund of Affiliated Cancer Hospital of Xinjiang Medical University, and Chinese Academy of Medical Sciences Innovation Fund for Medical Sciences (2021-I2M-1-004).

## Conflict of interest

The authors declare that the research was conducted in the absence of any commercial or financial relationships that could be construed as a potential conflict of interest.

## Publisher’s note

All claims expressed in this article are solely those of the authors and do not necessarily represent those of their affiliated organizations, or those of the publisher, the editors and the reviewers. Any product that may be evaluated in this article, or claim that may be made by its manufacturer, is not guaranteed or endorsed by the publisher.
