# Supplementary material for: Artificial intelligence in colposcopic examination: A promising tool to assist junior colposcopists
Source: Front Med (Lausanne). 2023 Mar 15;10:1060451. doi: 10.3389/fmed.2023.1060451 (PMC10088560; doi:10.3389/fmed.2023.1060451)
Supplement: Supplementary file 1 [file Data_Sheet_1.pdf]

Supplementary Material

1 Supplementary Figures

1.1 Supplementary Figure 1

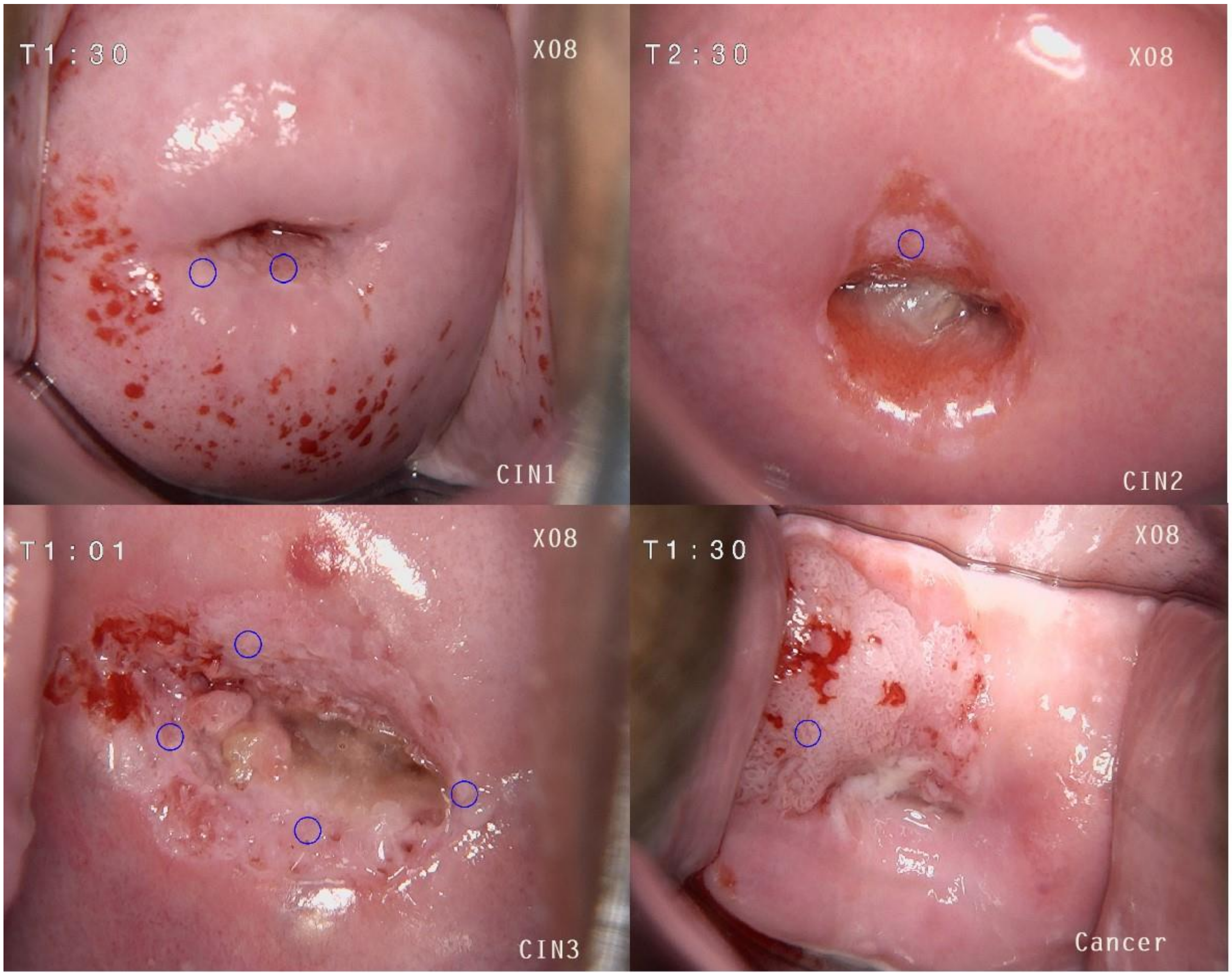

SupFig 1. Photos showing the biopsy sites indicated by CAIADS. The blue circles represent the indicated biopsy sites by CAIADS. Abbreviations: CAIADS, Colposcopic Artificial Intelligence Auxiliary Diagnostic System; CIN, cervical intraepithelial neoplasia; CIN1, CIN grade 1; CIN2, CIN grade 2; CIN3, CIN grade 3.

1.2 Supplementary Figure 2

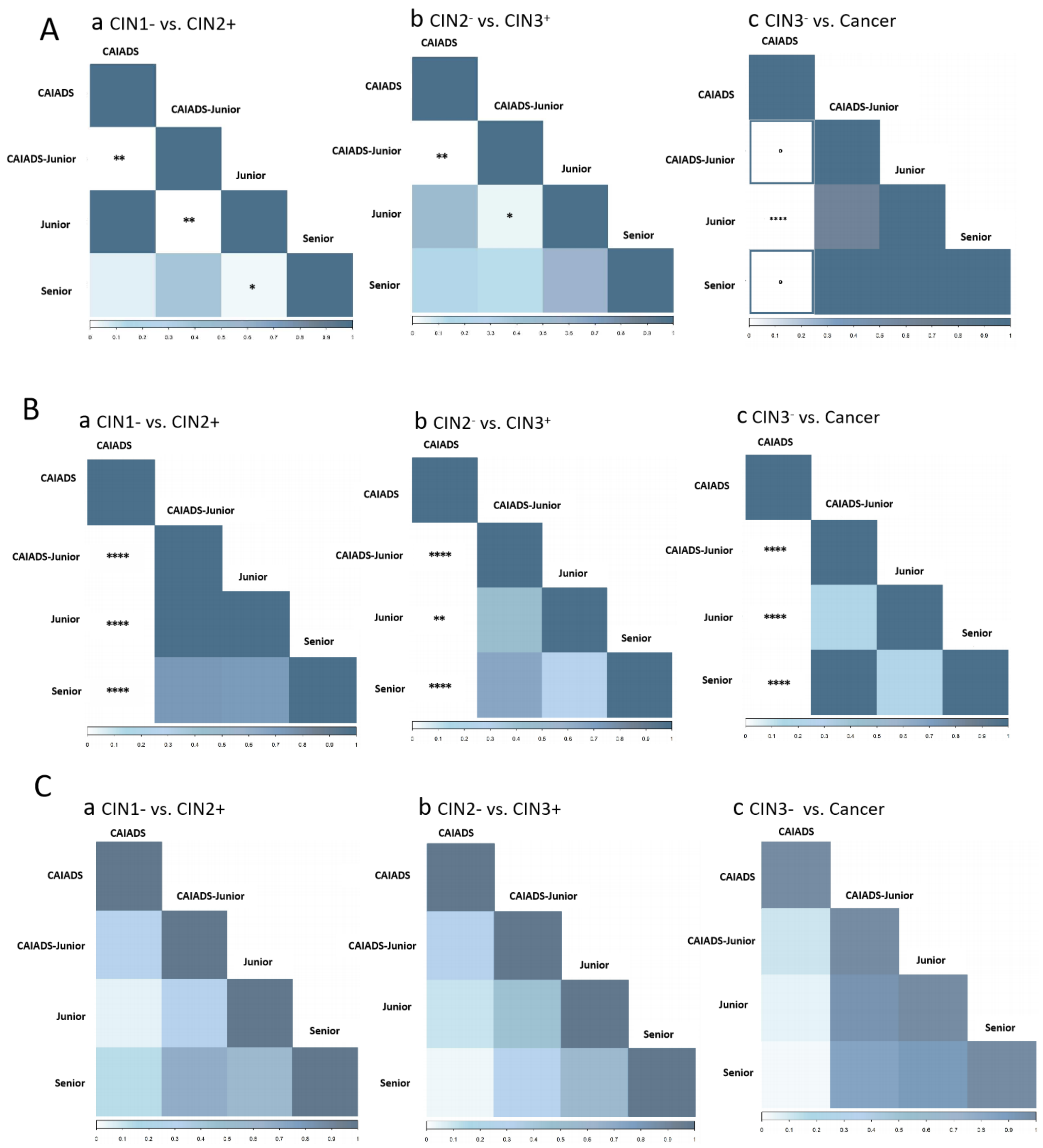

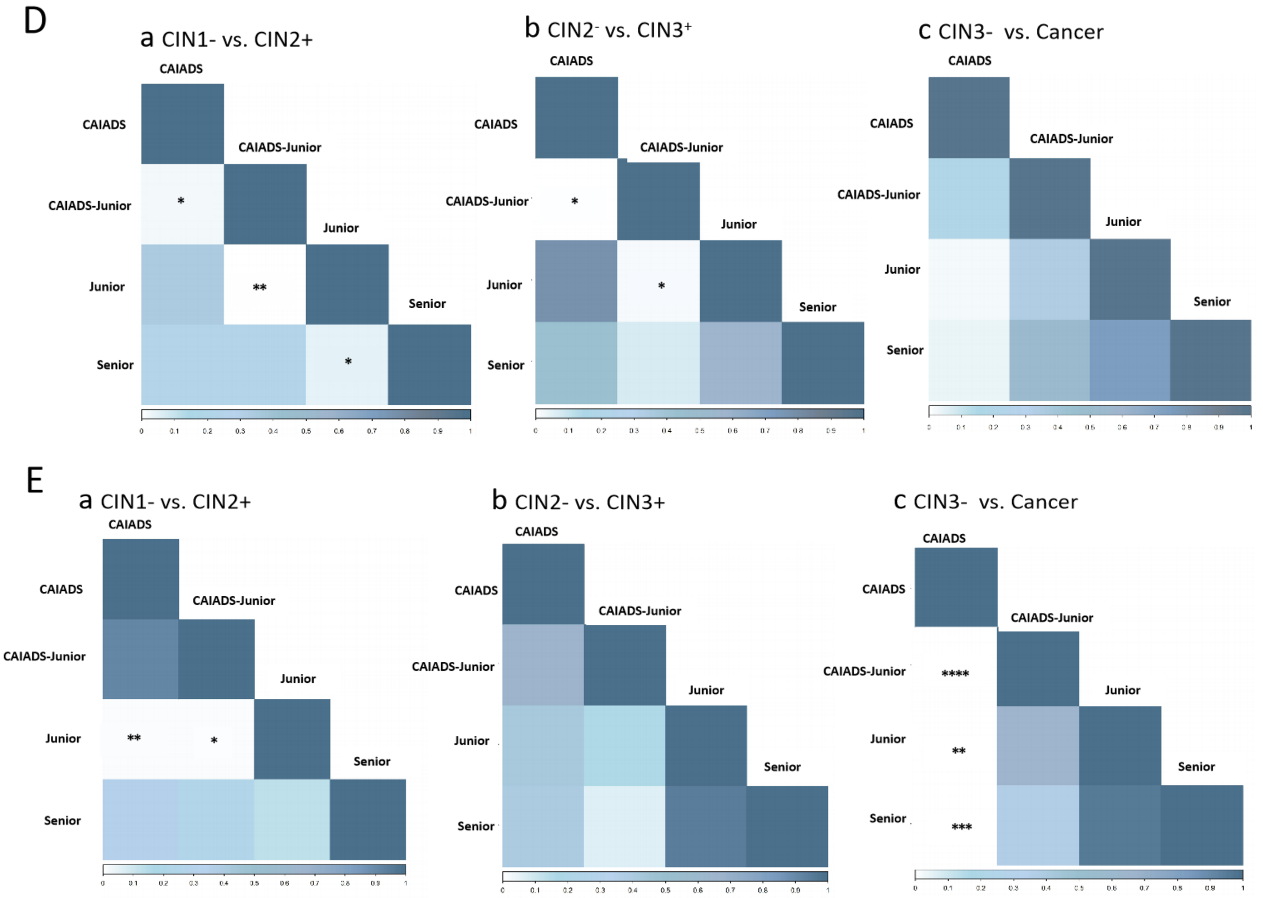

SupFig 2. The statistical differences of diagnostic performance between the subspecialists for detecting different histology results in the sensitivity (A), the specificity (B), the positive predictive value (C), the negative predictive value (D), and AUC (E). * $p < 0.05$, ** $p < 0.01$, *** $p < 0.001$, **** $p < 0.0001$. Abbreviations: CAIADS, Colposcopic Artificial Intelligence Auxiliary Diagnostic System; CAIADS-Junior, CAIADS-assisted junior colposcopist; CIN, cervical intraepithelial neoplasia; CIN1-, CIN grade 1 or less; CIN2-, CIN grade 2 or less; CIN3-, CIN grade 3 or less; CIN2+, CIN grade 2 or worse; CIN3+, CIN grade 3 or worse.

2 Supplementary Table 1

Supplement Table 1. Factors influencing the accuracy of CAIADS judgement

Characteristic	n(%)	Univariate Analysis OR (95% CI)	*p*	Multivariable Analysis OR(95% CI)	*p*
**Age**			**0.050**		0.135
[20-49]	200(54.6)	1	-	1	-
≥50	166(45.4)	0.50 (0.25-1.00)	-	0.50(0.20-1.24)	-
**Ethnicity**			0.911		0.333
Han	215(58.7)	1	-	1	-
Others[a]	151(41.3)	0.96 (0.49-1.90)	-	0.66(0.29-1.52)	-
**BMI**			0.169		0.227
[18.5-23.9]	193(52.7)	1	-	1	-
<18.5 or >23.9	173(47.3)	0.62 (0.31-1.22)	-	0.65(0.32-1.31)	-
**Education**			0.327		0.429
Blew middle school	137(37.4)	1	-	1	-
High school or above	229(62.6)	1.40 (0.71-2.76)	-	1.36(0.64-2.87)	-
**Number of parities**			0.416		0.109
≤1	189(51.6)	1	-	1	-
>1	177(48.4)	1.33 (0.67-2.62)	-	2.02(0.86-4.75)	-
**Stages of menopause**			0.117		0.727
Postmenopausal	113(30.9)	1	-	1	-
Pre-menopausal	253(69.1)	1.73 (0.87-3.44)	-	1.17(0.48-2.87)	-
**Cytology**			0.746		0.746
NILM	221(60.4)	1	-	1	-
ASC-US and LSIL	99(27.0)	0.84 (0.39-1.82)	0.658	0.82(0.37-1.83)	0.626
ASC-H, HSIL, and AGC	46(12.6)	0.70 (0.27-1.84)	0.471	0.70(0.26-1.90)	0.487
**HPV test**			0.773		0.529
Negative	58(15.8)	1	-	1	-
HPV 16/18 positive	164(44.8)	0.72 (0.26-2.03)	0.533	0.65(0.22-1.89)	0.430
Other high-risk subtypes positive	144(39.3)	0.88 (0.30-2.55)	0.808	0.97(0.32-2.94)	0.958
**Biopsy types**			0.237		0.266
Targeted biopsy	248(67.8)	1	-	1	-
Random biopsy	118(32.2)	1.60 (0.73-3.51)	-	1.58(0.71-3.54)	-

[a], Uighur, Kazak, Mongolian, Hui, Kirgiz; $p<0.05$; significant *p* values are bolded. Abbreviations: OR, odds ratio; CI, confidence interval; BMI, body mass index, kg/m^2; NILM, negative for intraepithelial lesion or malignancy; ASC-US, atypical squamous cells of undetermined significance; LSIL, low-grade squamous intraepithelial lesions; HSIL, high-grade squamous intraepithelial lesions; ASC-H, atypical squamous cells of undetermined significance cannot exclude HISL; AGC, atypical glandular cells; HPV, human papillomavirus.
